# Draft Genome Sequence of Bacillus sp. Strain M21, Isolated from the Arid Area of Matmata, Tunisia

**DOI:** 10.1128/genomeA.00323-18

**Published:** 2018-04-26

**Authors:** Zina Nasfi, Anja Poehlein, Henrik Harms, Emilie Goralski, Katja M. Fisch, Rolf Daniel, Gabriele M. König, Till F. Schäberle, Rafik Bachoual

**Affiliations:** a Biodiversity and Valorization of Bioresources in Arid Areas, Faculty of Sciences of Gabès, University of Gabès, Gabès, Tunisia; b Department of Genomics and Applied Microbiology and Göttingen Genomics Laboratory, Georg-August-University Göttingen, Göttingen, Germany; c Institute for Insect Biotechnology, Justus Liebig University Giessen, Giessen, Germany; d German Center for Infection Research (DZIF) Partner Site Cologne/Bonn, Bonn, Germany; e Institute for Pharmaceutical Biology, University of Bonn, Bonn, Germany

## Abstract

Bacillus sp. strain M21 was isolated from an environmental sample. In antibacterial screenings, the strain inhibited growth of Gram-positive and Gram-negative test strains. The genome was assembled into 69 contigs with a total size of 5.178 Mb. The strain contains at least nine biosynthetic gene clusters for the production of specialized metabolites.

## GENOME ANNOUNCEMENT

Gram-positive Bacillus species have the ability to form spores. This enables these strains to resist heat, which is a clear survival advantage at the surface in dry, hot regions, such as Matmata in southern Tunisia.

Bacillus sp. strain M21 was isolated during a bioproject aimed at evaluating the potential of bacterial strains from the arid zones of Tunisia to detect novel compounds with biological activities. It was retrieved from sandy sediment collected at a depth of 10 to 15 cm. The soil sample was transported immediately to the laboratory in a sterile flask, air dried for 2 h at 45°C, and sieved prior to use for isolation purposes. The soil sample was suspended in sterile water, and a dilution was plated onto LB agar (sodium chloride, 5 g/liter; tryptone, 10 g/liter; yeast extract, 5 g/liter; agar, 10 g/liter) plates. Antibacterial activities were assayed by a disc diffusion test using Mueller-Hinton (MH) agar plates (beef infusion solids, 2 g/liter; casein hydrolysate, 17.5 g/liter; starch, 1.5 g/liter; agar 17 g/liter). Bacillus sp. M21 was incubated for 24 h at 37°C in 20 ml LB broth. An aliquot (1%) was transferred into 100 ml LB broth and incubated at 37°C for 48 h. Cell-free supernatant (50 µl) was used to saturate a sterilized Whatman filter paper disc (6 mm), allowed to dry at room temperature, and placed onto MH agar plates, which were inoculated with 10^7^ CFU/ml of the test bacteria. The plates were incubated at 37°C for 24 h. Antibacterial activities were then evaluated by measuring the diameter of the inhibition zone around each paper disc. The potential of bacilli to produce natural products with antibacterial activity is well known (1).

A sample was prepared for sequencing by growing the strain aerobically at 37°C in LB medium for 24 h. Extraction of the genomic DNA was performed by using a kit (GenElute bacterial genomic DNA kit; Sigma-Aldrich). The extracted DNA was used to generate Illumina shotgun paired-end sequencing libraries, which were sequenced with a MiSeq instrument and the MiSeq reagent kit version 3, as recommended by the manufacturer (Illumina, San Diego, CA, USA). Quality filtering using Trimmomatic version 0.36 (2) resulted in 2,800,280 paired-end reads. The assembly was created with the SPAdes genome assembler software version 3.11.0 (3). The assembly resulted in 69 contigs (>500 bp) and an average coverage of 96-fold. The assembly was validated and the read coverage determined with Qualimap version 2.1 (4). The resulting draft genome is 5.178 Mbp in length, and the GC content is 35.17%. Automatic annotation and identification of rRNA and tRNA genes were performed using the software tool Prokka (5). The draft genome contains 11 rRNA genes, 90 tRNA genes, 3,673 protein-encoding genes with function prediction, and 1,535 genes coding for hypothetical proteins.

The tool antiSMASH 4.0.0 (6) was used for the *in silico* identification of biosynthetic gene clusters (BGCs) corresponding to the production of specialized metabolites, and nine putative BGCs were identified.

### Accession number(s).

This whole-genome shotgun project has been deposited at DDBJ/ENA/GenBank under the accession number PVNJ00000000. The version described in this paper is version PVNJ01000000.
